# Correlation of clinical characteristics between patients with seasonal influenza and patients infected by the wild type or delta variant of SARS-CoV-2

**DOI:** 10.3389/fpubh.2022.981233

**Published:** 2022-08-18

**Authors:** Jianguo Zhang, Xing Huang, Zhimin Tao

**Affiliations:** ^1^Department of Emergency Medicine, The Affiliated Hospital, Jiangsu University, Zhenjiang, China; ^2^Center for Evidence-Based and Translational Medicine, Zhongnan Hospital of Wuhan University, Wuhan, China; ^3^Jiangsu Provincial Key Laboratory of Medical Science and Laboratory Medicine, School of Medicine, Jiangsu University, Zhenjiang, China

**Keywords:** COVID-19, SARS-CoV-2, delta variant, influenza, recovery

## Abstract

**Background:**

We compared the clinical characteristics of the patients with COVID-19, infected by the wild type or delta variant of severe acute respiratory syndrome coronavirus 2 (SARS-CoV-2), in connection with those of patients with seasonal influenza, all in mild cases.

**Methods:**

We retrospectively studied 245 and 115 patients with mild COVID-19 infected by the wild type and the delta variant of SARS-CoV-2, respectively, with their demographic information, medical history, and laboratory data from hospital records, individually compared to 377 patients with mild seasonal influenza, before and after individual treatment.

**Results:**

Compared to the influenza cohort, the COVID-19 cohort or the COVID-19 delta variant cohort demonstrated younger median age, lower male ratio, and shorter duration from disease onset to hospitalization. Hypertension remained the top comorbidity among all cohorts. Based on patients' data upon hospitalization, the correlation of clinical characteristics between patients with influenza and those with the wild-type COVID-19 is greater than that between patients with influenza and those with the delta variant COVID-19. Individual treatment in each viral disease alleviated most hematological parameters, but some compromised biomarkers at the time of hospital discharge revealed persistent renal or myocardial impairment among patients with COVID-19 and influenza in recovery.

**Conclusion:**

Timely and proper treatment using broad-spectrum antibiotics and antiviral drugs could moderately alleviate the acute viremia and possible bacterial co-infection in patients with mild COVID-19 and influenza, followed by compromised recovery. To prepare for the flu season amid the COVID-19 pandemic, preventive and adequate immunizations of both flu and COVID-19 vaccines, as well as specific therapeutics to effectively reverse viral impairments, are in urgent need.

## Introduction

A novel coronavirus emerged in December 2019, and a global pandemic of pneumonia diseases began in March 2020 (1, 2). The disease was named COVID-19, and the pathogen responsible for COVID-19 was discovered as the severe acute respiratory syndrome coronavirus 2 (SARS-CoV-2) (3, 4). As of 19 June 2022, over 536 million cases of COVID-19 infection are confirmed globally, and the mortality rate of COVID-19 worldwide is estimated at ~1.2%, causing disastrous impacts on health, economic, and social sectors of human societies (5).

Severe acute respiratory syndrome coronavirus 2 shared a phylogenetic similarity to SARS-CoV with 79.6% nucleotide identity and to a lesser extent, Middle East respiratory syndrome coronavirus (MERS-CoV) with 51.8% identity (6, 7). The latter two pathogens caused 2002–2003 and 2012 regional outbreaks of acute respiratory diseases, each leading to a fatality of hundreds (8). In contrast, as SARS-CoV-2 itself rapidly evolves, five variants of concern (VOCs) have been designated insofar (9).

Similar to SARS-CoV, SARS-CoV-2 employed angiotensin-converting enzyme 2 (ACE2) in the host for viral entry, leading to intrapulmonary and extrapulmonary infections (6, 10). Many of the symptoms of patients with COVID-19 are common to those of patients with influenza, typified by predominant cough and fever (11). In the 2020–2021 winter, when pandemic COVID-19 collided with the flu season for the first time, influenza cases were unprecedentedly scarce and flu-caused hospitalization became the lowest ever recorded in North America, possibly due to the universal COVID-19 measures, such as travel restricting, social distancing, and mask wearing (12, 13). Comparatively, COVID-19 infections kept sweeping across the world at the same time, suggestive of its much higher infectivity than influenza. However, the loss of natural immunity developed for the circulating influenza virus in the past season may project a flu eruption in the incoming year (14). Even worse, it could pair with the rising SARS-CoV-2 variant infections.

Herein, we analyzed the clinical data of patients with mild COVID-19 infected by the wild type or delta variant of SARS-CoV-2 and compared them to those of patients with mild seasonal influenza, before and after individual treatment, to underline the differential characteristics of viral infection and recovery in COVID-19 from those in influenza. This study was to help understand the similarities and dissimilarities between COVID-19 and the flu and even between SARS-CoV-2 infections by different variants.

## Methods

### Study design

The study was approved by The First People's Hospital of Jiangxia District (TFPHJD) in Wuhan, The Third People's Hospital of Yangzhou City (TTPHYC), and The Affiliated Hospital of Jiangsu University (TAHJU) in Zhenjiang, China, respectively. A total of 245 patients with laboratory-confirmed COVID-19 were hospitalized in the non-intensive care unit (non-ICU) isolation wards in TFPHJD between 1 February 2020 and 15 April 2020. In a different cohort, 115 unvaccinated COVID-19 patients were infected with the delta variant of SARS-CoV-2 and admitted by TTPHYC in August 2021. All patients with COVID-19 tested negative for influenza, but whether they have been previously vaccinated with flu shots remained unknown. In parallel, 377 patients with influenza were diagnosed and admitted at TAHJU from January 2017 to September 2020, where no patients with COVID-19 had been reported. No patients with influenza have been immunized with flu shots or COVID-19 vaccines. For all cohorts, patient information remained anonymous, and written consent of patients was waived by the Ethics Commission of TAHJU, TTPHYC, or TFPHJD, correspondingly. Exclusion criteria include patients below 18 years, and patients with pregnancy, terminal illness, immunodeficiency, or congenital heart/renal diseases.

### Patient procedure

A total of 245 patients with COVID-19 were admitted at TFPHJD, following a standard procedure, as previously reported (15). The confirmed patients were treated with antiviral drugs, including oseltamivir, arbidol, and ribavirin (16, 17). For 115 patients infected by the delta variant of SARS-CoV-2, they were previously unvaccinated and treated with the Chinese traditional medicine and antibiotics (ceftazidime and levofloxacin) if the bacterial infection was assessed. In contrast, patients with influenza were diagnosed using a detection kit of serum IgM antibodies against respiratory viruses based on an indirect immunofluorescence assay (EUROIMMUN, Germany). Among 377 patients with influenza, 355 (94.2%) patients were infected with influenza A virus, 305 (80.9%) patients were infected with influenza B, and 283 (75.1%) patients were co-infected. Patients with influenza were hospitalized at TAHJU, where oxygen therapy was applied along with ribavirin or oseltamivir antiviral treatment. None of the patients with COVID-19 or influenza included in this study developed any severe, critically ill, or fatal conditions. A blood cell analysis was performed using an automated hematology analyzer (SYSMEX 800i, Japan; Mindray BC-5300, China), and the biochemical indicators were analyzed (Toshiba TAB2000, Japan; Beckman AU5800, USA; Roche Cobas 6000 Analyzer, Switzerland).

### Data collection and analysis

Demographic data, medical history, and clinical characteristics of patients with COVID-19 or influenza were obtained at TAHJU, TTPHYC, and TFPHJD. All blood parameters were collected from patients upon hospital admission, and for blood testing after treatment, we adopted the last dataset of patients before they were discharged from the hospital. The categorical variables were described as frequency rates and percentages, and continuous variables were applied to describe the median and quartile range (IQR) values. A comparison of continuous variables between the two groups was analyzed with the Mann–Whitney test. The Chi-square test was used to compare the proportion of categorical variables. Variables according to their clinical relevance and statistical significance in univariate analysis (*p* < 0.05) were included in the multivariate logistic regression analysis, which was further performed to explore the independent risk factors associated. All statistical analyses were performed using the statistical package for social sciences (SPSS) version 13.0 software (SPSS Inc.). A two-sided α of <0.05 was considered statistically significant.

## Results

### Comparison of baseline information and clinical symptoms between the patients with seasonal influenza and the patients with COVID-19 infected by the wild type or the delta variant of SARS-CoV-2

A total of 737 patients were reported in this study, including 245 patients with COVID-19, 115 patients with COVID-19 infected by the delta variant of SARS-CoV-2 (denoted as COVID-19 Δ), and 377 patients with seasonal influenza. In the COVID-19 and COVID-19 Δ cohort, the median age of patients was 51.0 (IQR 39.0–63.0) and 63.0 (IQR 35.0–72.0), 48.6 and 42.6% of them were men, and the time between disease onset and hospital admission spanned 4.0 days (IQR 3.0–5.0) and 2.0 days (IQR 1.0–4.0), respectively. In comparison with either COVID-19 or COVID-19 Δ cohort, the influenza cohort exhibited a much higher median age of patients and male ratio and a longer duration from disease onset to hospitalization. For the portion of patients with a smoking history, the influenza cohort appeared similar to the COVID-19 cohort but higher than the COVID-19 Δ cohort.

Regarding the leading comorbidities among the hospitalized patients, patients with influenza had the most occurrence of co-existing hypertension and the least occurrence of comorbid diabetes, whereas influenza patients with bronchitis showed much higher frequency than patients with COVID-19. For the major comorbidity in COVID-19 and COVID-19 Δ cohorts, hypertension ranked the top one followed by diabetes. In addition, COVID-19 Δ cohort demonstrated a marginal portion of patients with bronchitis, possibly related to the small ratio of patients with a smoking history. Hence, hypertension, being the top comorbidity, put patients at the highest risk for both influenza and COVID-19 infection, while diabetes and cardiovascular diseases made up substantial risk factors to have an adverse impact on patients with influenza and COVID-19.

At the disease onset, COVID-19 illness manifested the common clinical symptoms as follows (Table 1): cough (85.7%), fever (83.3%), fatigue (38.4%), chest pain (24.9%), abdominal pain (15.5%), diarrhea (15.1%), and vomiting (9.8%), each with higher frequency than that in influenza cohort, except that influenza patients with symptoms of expectoration or dyspnea showed more incidence. Thus, the patients with COVID-19 were more likely to show initial symptoms compared with those with influenza. Reversely, the COVID-19 Δ cohort revealed much less incidence of symptoms when compared to the influenza cohort in general (except for fatigue and diarrhea), showing distinctive profiles from the COVID-19 cohort. Notably, a fair number of patients with mild viral infections by the influenza virus, the wild-type, and the delta variant SARS-CoV-2 experienced no fever or cough.

**Table 1:** Demographic data, medical history, and clinical symptoms of 377 patients with influenza vs. 245 patients with COVID-19 infected with the wild-type SARS-CoV-2 or 115 patients with COVID-19 infected with the delta variant SARS-CoV-2 upon hospital admission.

	***p***	**COVID-19 (*n* = 245)**	**Influenza (*n* = 377)**	**COVID-19 Δ (*n* = 115)**	***p* ^Δ^**
Age	<0.0001	51.0 (39.0–63.0)	69.0 (57.0–77.0)	63.0 (35.0–72.0)	0.0001
Gender, male	<0.001	119 (48.6%)	236 (62.6%)	49 (42.6%)	0.0001
Onset to hospitalization, day	<0.0001	4.0 (3.0–5.0)	5.0 (3.5–6.0)	2.0 (1.0–4.0)	<0.0001
Smoking history	0.735	62 (25.3%)	100 (26.5%)	13 (11.3%)	<0.001
**Comorbidity**					
Hypertension	<0.0001	46 (18.8%)	150 (39.8%)	36 (31.3%)	0.101
Bronchitis	0.067	16 (6.5%)	41 (10.9%)	1 (0.9%)	<0.001
Cardiovascular diseases	0.002	8 (3.3%)	38 (10.1%)	13 (11.3%)	0.706
Diabetes	0.019	27 (11.0%)	22 (5.8%)	17 (14.8%)	0.002
**Symptoms**					
Fever	<0.0001	204 (83.3%)	243 (64.5%)	43 (37.4%)	<0.0001
Cough	<0.0001	210 (85.7%)	240 (63.7%)	61 (53.0%)	0.041
Expectoration	<0.0001	34 (13.9%)	238 (63.1%)	17 (14.8%)	<0.0001
Dyspnea	<0.0001	14 (5.7%)	64 (17.0%)	1 (0.9%)	<0.0001
Chest pain	<0.0001	61 (24.9%)	16 (4.2%)	4 (3.5%)	1.000
Abdominal pain	<0.0001	38 (15.5%)	12 (3.2%)	1 (0.9%)	0.317
Fatigue	<0.0001	94 (38.4%)	11 (2.9%)	28 (24.3%)	<0.0001
Diarrhea	<0.0001	37 (15.1%)	7 (1.9%)	7 (6.1%)	0.017
Vomiting	<0.0001	24 (9.8%)	7 (1.9%)	1 (0.9%)	0.688

p and pΔ denote the statistical significance of the observed difference between the COVID-19 and influenza cohorts, and that between the COVID-19 Δ and influenza cohorts, respectively.

### Comparison of blood parameters between the patients with seasonal influenza and the patients infected by the wild type or the delta variant of SARS-CoV-2

The laboratory blood tests of patients upon their hospitalization were performed, and typical parameters indicating hematological, metabolic, and organ functions are listed in Table 2. Given the abnormality in cell number and the patient ratio with abnormal cell counts, lymphocytopenia was similarly severe in all cohorts, tracing considerable viral infection. Compared to the COVID-19 or COVID-19 Δ cohort, the influenza cohort exhibited more severe leukocytosis, neutrophilia, and anemia, but less or comparably severe thrombocytopenia. Of them, almost half portion of all patients showed anemia, reflected by abnormally low levels of red blood cell (RBC), hemoglobin, and hematocrit. However, anemic conditions were significantly mitigated in the COVID-19 Δ cohort. For coagulation factors, all patients demonstrated severe coagulopathy. In comparison with the COVID-19 cohort, the influenza cohort possessed substantially reduced prothrombin time and aPTT, but increased thrombin time and fibrinogen level, and greatly elevated D-dimer concentration; in comparison with the COVID-19 Δ cohort, the influenza cohort owned similar prothrombin time and reduced aPTT and thrombin time, but augmented fibrinogen and D-dimer levels. D-dimer was widely applied as an indicator for thrombotic disorders, and it was observed that patients with mild COVID-19 might exhibit a less severe thrombotic state than those with influenza, and this coagulopathy was even alleviated in patients with mild COVID-19 infected with the delta variant SARS-CoV-2 where thrombocytopenia was the worst among all cohorts.

**Table 2:** Laboratory blood tests of the influenza cohort vs. the COVID-19 cohort or COVID-19 Δ cohort upon hospital admission.

	**Normal range**	***p***	**COVID-19 (*n* = 245)**	**Influenza (*n* = 377)**	**COVID-19 Δ (*n* = 115)**	***p*Δ**
**Blood cell count**						
WBCs, × 10^9^/L	3.5–9.5	<0.0001	6.0 (4.7–7.5)	7.4 (5.5–10.1)	4.8 (3.8–5.7)	<0.0001
>9.5		<0.0001	23 (9.4%)	103 (27.3%)	1 (0.9%)	<0.0001
Neutrophils, × 10^9^/L	1.8–6.3	<0.0001	4.3 (2.8–5.9)	5.5 (3.7–8.0)	3.0 (2.1–3.9)	<0.0001
>6.3		<0.0001	43 (17.6%)	144 (38.2%)	5 (4.3%)	<0.0001
Lymphocytes, × 10^9^/L	1.1–3.2	0.688	1.1 (0.8–1.6)	1.1 (0.8–1.6)	1.2 (0.9–1.5)	0.588
<1.1		0.582	116 (47.3%)	170 (45.1%)	56 (48.7%)	0.497
RBCs, × 10^12^/L	4.3–5.8	0.025	4.3 (3.9–4.6)	4.2 (3.7–4.6)	4.4 (4.0–4.9)	<0.001
<4.3		0.033	125 (51.0%)	225 (59.7%)	47 (40.9%)	<0.001
Hemoglobin, g/L	130–175	0.137	128.0 (115.0–140.0)	127.0 (111.5–138.0)	135.0 (122.0–143.0)	<0.0001
<130		0.301	132 (53.9%)	219 (58.1%)	49 (42.6%)	0.004
HCT, %	40–50	0.645	37.5 (34.3–40.8)	38.0 (33.9–41.8)	39.1 (36.0–42.6)	0.040
<40		0.381	165 (67.3%)	241 (63.9%)	64 (55.7%)	0.110
Platelets, × 10^9^/L	125–350	0.367	197.0 (151.0–258.0)	206.0 (154.0–268.0)	155.0 (130.0–194.0)	<0.0001
<125		0.830	28 (11.4%)	41 (10.9%)	24 (20.7%)	0.006
**Coagulation factors**						
Prothrombin time, s	9–13	<0.0001	13.4 (12.6–14.0)	12.1 (11.3–14.1)	12.0 (11.6–12.5)	0.075
>13		<0.0001	152 (62.0%)	135 (35.8%)	14 (12.2%)	<0.0001
INR	0.8–1.2	0.168	1.07 (1.01–1.13)	1.03 (0.97–1.17)	1.05 (1.01–1.09)	0.749
>1.2		0.0001	23 (9.4%)	80 (21.2%)	4 (3.5%)	<0.0001
aPTT, s	23.3–32.5	<0.0001	30.1 (28.2–31.5)	27.4 (24.7–31.3)	30.8 (28.3–33.6)	<0.0001
>32.5		0.061	31 (12.7%)	69 (18.3%)	35 (30.4%)	0.005
Thrombin time, s	14–21	<0.0001	15.9 (15.0–17.0)	17.7 (16.7–19.5)	18.2 (17.4–18.9)	0.046
>21		<0.0001	0 (0)	82 (21.8%)	4 (3.5%)	<0.0001
Fibrinogen, g/L	2–4	<0.0001	3.5 (2.6–4.3)	4.2 (3.1–5.3)	3.2 (2.6–3.9)	<0.0001
>4		<0.0001	83 (33.9%)	204 (54.1%)	24 (20.9%)	<0.0001
D-dimer, mg/L	<0.55	<0.0001	0.62 (0.24–1.22)	1.05 (0.48–2.42)	0.39 (0.24–0.57)	<0.0001
>0.55		<0.0001	131 (53.5%)	266 (70.6%)	29 (25.2%)	<0.0001
**Metabolic panel**						
PCT, ng/mL	<0.1	<0.0001	1.1 (0.5–1.6)	6.2 (2.9–12.9)	0.04 (0.03–0.05)	<0.0001
>0.1		0.141	239 (97.6%)	359 (95.2%)	10 (8.7%)	<0.0001
CRP, mg/ L	0–10	0.361	22.7 (12.8–55.8)	22.5 (7.7–77.5)	10.9 (3.0–29.2)	<0.0001
>10		<0.0001	212 (86.5%)	271 (71.9%)	60 (52.2%)	<0.0001
ALT, U/L	9–50	<0.0001	24.0 (17.9–35.9)	36.1 (20.1–70.5)	20.0 (13.1–31.5)	<0.0001
>50		<0.0001	22 (9.0%)	148 (39.3%)	12 (10.4%)	<0.0001
AST, U/L	15–40	<0.0001	22.7 (14.7–38.1)	39.5 (19.2–72.5)	23.6 (19.3–38.2)	<0.0001
>40		<0.0001	53 (21.6%)	185 (49.41%)	27 (23.5%)	<0.0001
ALP, U/L	32–126	<0.001	66.0 (54.0–91.0)	76.0 (59.0–113.0)	84.0 (72.0–105.0)	0.013
>126		0.001	23 (9.4%)	74 (19.6%)	13 (11.3%)	0.041
BUN, mmol/L	2.86–8.2	<0.0001	4.4 (3.4–5.5)	6.8 (4.4–10.8)	4.6 (3.7–5.7)	<0.0001
>8.2		<0.0001	19 (7.8%)	145 (38.5%)	10 (8.7%)	<0.0001
Albumin, g/L	40–55	<0.0001	33.5 (29.6–37.4)	31.5 (27.5–35.9)	45.4 (42.7–48.2)	<0.0001
<40		0.001	206 (84.1%)	348 (92.3%)	13 (11.3%)	<0.0001
CPK, U/L	38–174	<0.0001	62.0 (47.0–90.0)	78.0 (55.0–128.5)	94.0 (60.0–148.0)	0.129
>174		0.091	30 (12.2%)	65 (17.2%)	21 (18.3%)	0.781
CK–MB, U/L	0–25	<0.0001	55.8 (34.9–77.1)	21.9 (13.1–45.7)	13.3 (10.6–15.7)	<0.0001
>25		<0.0001	209 (85.3%)	178 (47.2%)	9 (7.8%)	<0.0001
LDH, U/L	80–285	<0.0001	366.0 (225.5–530.0)	264.0 (182.0–361.5)	201.0 (177.0–247.0)	<0.0001
>285		<0.0001	154 (62.9%)	162 (43.0%)	18 (15.7%)	<0.0001
Potassium, mmol/L	3.5–5.3	<0.0001	4.2 (3.7–4.6)	3.8 (3.5–4.3)	3.7 (3.4–4.1)	0.015
<3.5		0.006	38 (15.5%)	93 (24.7%)	34 (29.6%)	0.294
Sodium, mmol/L	137–147	<0.0001	142.7 (137.1–147.1)	138.2 (134.7–141.4)	137.0 (135.0–139.0)	0.017
<137		<0.0001	61 (24.9%)	153 (40.6%)	44 (38.3%)	0.656
Total calcium, mmol/L	2.08–2.6	<0.0001	1.78 (1.53–2.02)	2.11 (2.00–2.25)	2.28 (2.20–2.36)	<0.0001
<2.08		<0.0001	200 (81.6%)	160 (42.4%)	4 (3.5%)	<0.0001

p and pΔ denote each statistical significance of the observed difference between two cohorts.

WBC, white blood cell; RBC, red blood cell; HCT, hematocrit; INR, international normalized ratio; aPTT, activated partial thromboplastin time; PCT, procalcitonin; CRP, c-reactive protein; ALT, alanine aminotransferase; AST, aspartate aminotransferase; ALP, alkaline phosphatase; BUN, blood urea nitrogen; CPK, creatine phosphokinase; CK-MB, creatine kinase isoenzyme; LDH, lactate dehydrogenase.

The abnormality in blood cell counts and coagulation factors suggested viral/bacterial (co)infection and the induced inflammatory response, further confirmed by the heightened levels of c-reactive protein (CRP) and procalcitonin (PCT) in most patients of COVID-19 and influenza cohorts. Markedly, the COVID-19 Δ cohort showed surprisingly low levels of PCT and CRP, implying a mild infection with a weakened inflammatory response for hospitalized patients upon admission. Simultaneously, compared to those in the COVID-19 cohort, in terms of testing values and ratio of patients with abnormal testing values, the levels of alanine aminotransferase (ALT), aspartate aminotransferase (AST), alkaline phosphatase (ALP), and blood urea nitrogen (BUN) in the influenza cohort were monitored to be much higher, while the concentrations of albumin, creatine kinase isoenzymes (CK-MB), and lactate dehydrogenase (LDH) were demonstrated to be much lower, signifying the increased risks of hepatic/renal disorders in patients with mild influenza but heightened risks of adverse cardiac events in those with mild COVID-19. In parallel, the COVID-19 Δ cohort displayed a declining impact on major organs, reflected by the fact that most biochemical biomarkers showed lessened values when compared to those in the influenza cohort, such as ALT, AST, BUN, CK-MB, and LDH, showing the diminished viremia of the delta variant SARS-CoV-2. Besides, electrolyte imbalance was found common in all COVID-19, influenza, and COVID-19 Δ cohorts, as traces of hypokalemia, hyponatremia, and hypocalcemia occurred in a portion of patients.

### Correlations of clinical characteristics between the patients with seasonal influenza and the patients infected by the wild type or the delta variant of SARS-CoV-2

**Figure 1:**
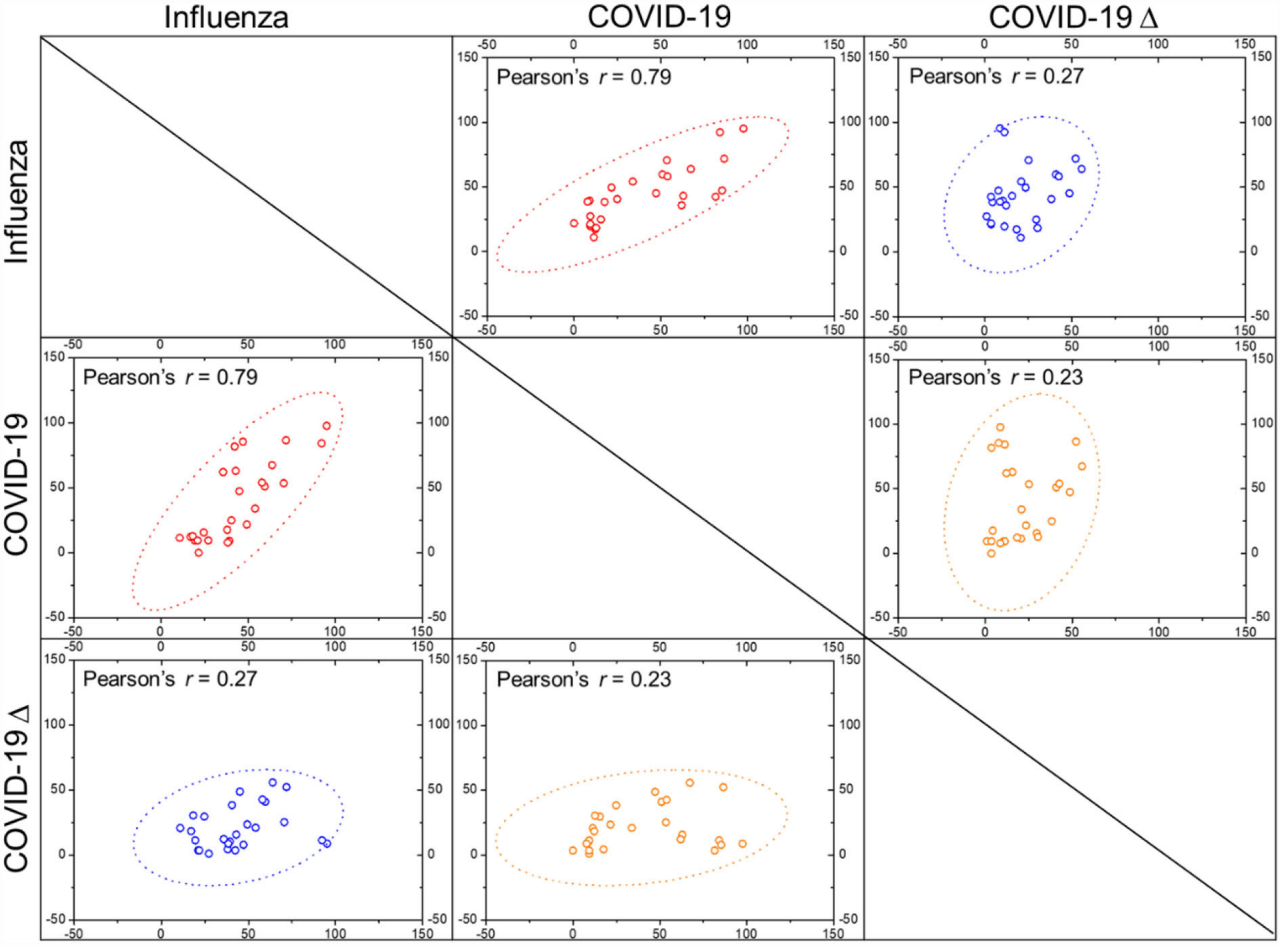
Pearson's correlation coefficients (*r* values) were calculated between any two cohorts as indicated, with dotted ellipse showing confidence, by using the frequencies of patients with deranged blood characters (scattered dots) as indicated in Table 2.

Variables with significant differences between the influenza and COVID-19 cohorts were further collected for multivariate logistic regression analysis (Table 3). It is derived that age, time from disease onset to hospitalization, diabetes and cardiovascular disease comorbidities, symptoms of fever and cough, metabolic biomarkers of BUN, CK-MB, and LDH, and electrolyte balances of K^+^ and Ca^2+^ represent the independent risk factors to differentiate patients with influenza from patients with COVID-19 based on patients' blood parameters. Similarly, variables with significant differences between the influenza and COVID-19 Δ cohorts were also analyzed using multivariate logistic regression (Table 4). As a result, high age, fever, and aberrations in cell counts of WBC, RBC, and platelets and levels of D-dimer, PCT, CK-MB, and Ca^2+^ constitute the independent risk factors to differentiate patients with influenza from patients with COVID-19 Δ. In parallel, we performed Pearson's correlation analysis on any of two cohorts, using the frequencies of patients with abnormal values of blood parameters, to evaluate the correlations of clinical characteristics between the influenza cohort and the COVID-19 or COVID-19 Δ cohort. Results are shown in Figure 1. Pearson's correlation coefficient (PCC) between the influenza cohort and the COVID-19 cohort read 0.79 (with significance <0.00001), higher than the PCC between the influenza cohort and the COVID-19 Δ cohort which read 0.27 (with a significance of 0.179). In addition, the PCC between the wild type and delta variant COVID-19 cohorts was 0.23 (with a significance of 0.254).

**Table 3:** Variables (*p* < 0.05) with clinical relevance were performed using multivariate logistic regression analysis to explore the independent risk factors associated with differences between the influenza cohort and the COVID-19 cohort.

**Variables**	***p***	**Hazardous ratio**	**95% confidence interval**
Age	<0.001	0.953	0.937–0.969
Male ratio	0.548	1.187	0.679–2.074
Disease onset to hospitalization	<0.001	0.636	0.534–0.758
Hypertension	0.075	0.562	0.297–1.060
Diabetes	0.007	5.227	1.577–17.322
Cardiovascular diseases	0.001	0.137	0.042–0.442
Cough	<0.001	4.066	2.128–7.770
Fever	<0.001	4.591	2.411–8.741
WBC	0.189	0.5	0.178–1.406
Neutrophil	0.121	0.501	0.209–1.201
RBC	0.293	0.74	0.423–1.297
D-dimer	0.942	1.022	0.577–1.809
CRP	0.154	1.629	0.833–3.186
BUN	<0.001	0.054	0.024–0.120
CK-MB	<0.001	12.279	6.422–23.478
LDH	0.022	1.914	1.099–3.333
Hypokalemia	0.010	0.392	0.192–0.799
Hypocalcemia	<0.001	7.107	3.876–13.032

**Table 4:** Variables (*p* < 0.05) with clinical relevance were performed using multivariate logistic regression analysis to explore the independent risk factors associated with differences between the influenza cohort and the COVID-19 Δ cohort.

**Variables**	***p***	**Hazardous ratio**	**95% confidence interval**
Age	0.108	0.969	0.932–1.007
Male ratio	0.085	3.013	0.859–10.562
Disease onset to hospitalization	0.208	0.857	0.674–1.090
Smoking	0.975	0.973	0.179–5.285
Diabetes	0.363	2.284	0.386–13.523
Cough	0.103	3.502	0.775–15.834
Fever	0.006	0.168	0.047–0.607
WBC	<0.001	0.001	0.000–0.057
Neutrophil	0.397	2.18	0.359–13.233
RBC	0.045	0.264	0.072–0.970
Platelets	0.001	14.171	2.771–72.463
D-dimer	0.002	0.106	0.025–0.448
PCT	<0.001	0.001	0.000–0.009
CRP	0.716	1.266	0.355–4.515
BUN	0.590	1.485	0.352–6.260
CK-MB	0.004	0.094	0.019–0.464
LDH	0.913	0.925	0.230–3.731
Hypocalcemia	0.018	0.102	0.015–0.678

For patients with mild infection in the COVID-19 cohort, they were treated with antibiotics including sulperazone and linezolid, and antiviral drugs including oseltamivir, arbidol, and ribavirin. After treatment, most hematological parameters were ameliorated, such as lymphocytopenia, thrombocytopenia, and elevated levels of D-dimer, CRP, PCT, ALT, AST, and ALP. Among those, some myocardial biomarkers were substantially improved, including CK-MB and LDH, suggesting a decent cardiac recovery (Table 5). In addition, anemic conditions and hypokalemic and hyponatremic disturbances remained not improved after treatment. Nonetheless, several disorders, including leukocytosis, neutrophilia, and heightened BUN level, became even worse after treatment, pointing to persistent blood infection after viral clearance. Concurrently, in the influenza cohort, patients were treated using oxygen therapy, along with ribavirin or oseltamivir. As a result, most blood parameters became much better, although anemia, abnormally high levels of BUN and CPK, and hypocalcemia stayed statistically unchanged and the level of CK-MB still climbed after treatment, denoting the sustained myocardial impairment in the patients with mild influenza upon hospital discharge. In the COVID-19 Δ cohort, the patients were treated with traditional Chinese medicine and antibiotics (ceftazidime and levofloxacin) if the bacterial coinfection was assessed. Post-treatment, except for deterioration of leukocytosis and anemia, major blood characters were restored at the time of hospital discharge. Especially, recovery in the main infection indicators (e.g., lymphocytopenia, thrombocytopenia, elevated CRP, and hypokalemia) and myocardial biomarkers (e.g., CPK, CK-MB, and LDH) exhibited good convalescence.

**Table 5:** Blood parameters of patients in the COVID-19 cohort, the influenza cohort, and the COVID-19 Δ cohort, before and after individual treatment.

	**Normal range**	**COVID-19 (*****n*** = **245)**			**Influenza (*****n*** = **377)**			**COVID-19** Δ **(*****n*** = **115)**		
		**Before treatment**	**After treatment**	***p*-value**	**Before treatment**	**After treatment**	***p*-value**	**Before treatment**	**After treatment**	***p*-value**
WBCs, × 10^9^/L	3.5–9.5	6.0 (4.7–7.5)	6.3 (5.3–7.4)	0.024	7.4 (5.5–10.1)	6.5 (5.2–8.4)	<0.0001	4.8 (3.8–5.7)	5.6 (4.8–6.7)	<0.0001
Neutrophils, × 10^9^/L	1.8–6.3	4.3 (2.8–5.9)	5.0 (3.8–6.2)	0.002	5.5 (3.7–8.0)	4.8 (3.4–6.6)	<0.0001	3.0 (2.1–3.9)	3.1 (2.6–3.7)	0.537
Lymphocytes, × 10^9^/L	1.1–3.2	1.1 (0.8–1.6)	1.4 (1.1–1.8)	<0.0001	1.1 (0.8–1.6)	1.3 (0.9–1.8)	0.013	1.2 (0.9–1.5)	1.7 (1.4–2.2)	<0.0001
RBCs, × 10^12^/L	4.3–5.8	4.3 (3.9–4.6)	4.3 (4.0–4.7)	0.146	4.2 (3.7–4.6)	4.2 (3.7–4.6)	0.512	4.4 (4.0–4.9)	4.2 (3.7–4.6)	<0.0001
Hemoglobin, g/L	130–175	128.0 (115.0–140.0)	136.0 (121.0–146.0)	<0.0001	127.0 (111.5–138.0)	126.0 (109.0–139.0)	0.953	135.0 (122.0–143.0)	127.0 (114.0–139.0)	<0.0001
HCT, %	40–50	37.5 (34.3–40.8)	39.3 (36.1–42.5)	<0.0001	38.0 (33.9–41.8)	37.9 (32.9–41.1)	0.010	39.1 (36.0–42.6)	37.5 (33.4–40.7)	<0.0001
Platelets, × 10^9^/L	125–350	197.0 (151.0–258.0)	237.0 (186.0–282.0)	<0.0001	206.0 (154.0–268.0)	217.0 (168.5–278.5)	0.001	155.0 (130.0–194.0)	233.0 (185.0–299.0)	<0.0001
Prothrombin time, s	9–13	13.4 (12.6–14.0)	12.7 (11.8–13.7)	<0.0001	12.1 (11.3–14.1)	11.6 (10.9–12.4)	<0.0001	12.0 (11.6–12.5)	11.3 (10.9–11.8)	<0.001
INR	0.8–1.2	1.07 (1.01–1.13)	1.13 (1.04–1.24)	0.315	1.03 (0.97–1.17)	1.05 (0.97–1.15)	0.316	1.05 (1.01–1.09)	0.97 (0.93–1.03)	0.002
aPTT, s	23.3–32.5	30.1 (28.2–31.5)	29.9 (27.7–31.5)	0.082	27.4 (24.7–31.3)	27.3 (24.9–29.8)	0.008	30.8 (28.3–33.6)	27.9 (25.8–30.4)	<0.0001
Thrombin time, s	14–21	15.9 (15.0–17.0)	15.6 (14.3–16.6)	0.078	17.7 (16.7–19.5)	17.5 (16.4–18.9)	<0.0001	18.2 (17.4–18.9)	18.1 (17.4–19.0)	0.961
Fibrinogen, g/L	2–4	3.5 (2.6–4.3)	3.7 (3.2–4.3)	0.001	4.2 (3.1–5.3)	3.5 (2.8–4.1)	<0.0001	3.2 (2.6–3.9)	3.3 (2.6–3.8)	0.466
D–dimer, mg/L	<0.55	0.62 (0.24–1.22)	0.47 (0.21–0.73)	<0.0001	1.05 (0.48–2.42)	0.62 (0.36–1.16)	<0.0001	0.39 (0.24–0.57)	0.42 (0.23–0.57)	0.384
CRP, mg/ L	0–10	22.7 (12.8–55.8)	5.4 (1.8–13.6)	<0.0001	22.5 (7.7–77.5)	10.5 (2.5–36.5)	<0.0001	10.9 (3.0–29.2)	1.7 (0.6–6.9)	<0.0001
PCT, ng/mL	<0.1	1.1 (0.5–1.6)	0.4 (0.2–1.0)	0.041	6.2 (2.9–12.9)	1.5 (0.7–4.6)	<0.0001	0.04 (0.02–0.05)	0.04 (0.03–0.05)	0.304
ALT, U/L	9–50	24.0 (17.9–35.9)	22.2 (17.5–28.1)	<0.0001	36.1 (20.1–70.5)	26.2 (15.5–46.0)	<0.001	20.0 (13.1–31.5)	24.8 (17.0–43.6)	<0.001
AST, U/L	15–40	22.7 (14.7–38.1)	16.6 (13.1–25.9)	<0.0001	39.5 (19.2–72.5)	24.6 (16.2–42.9)	0.056	23.6 (19.3–38.2)	22.7 (17.2–37.1)	0.200
ALP, U/L	32–126	66.0 (54.0–91.0)	62.0 (47.0–83.0)	0.001	76.0 (59.0–113.0)	72.2 (53.0–94.0)	0.052	84.0 (72.0–105.0)	83.0 (69.0–96.0)	0.285
BUN, mmol/L	2.86–8.2	4.4 (3.4–5.5)	6.7 (4.5–9.4)	<0.0001	6.8 (4.4–10.8)	6.5 (4.6–10.6)	0.928	4.6 (3.7–5.7)	4.8 (3.9–6.1)	0.379
Albumin, g/L	40–55	33.5 (29.6–37.4)	38.3 (34.6–44.6)	<0.0001	31.5 (27.5–35.9)	35.7 (28.8–38.9)	<0.0001	45.4 (42.7–48.2)	41.7 (38.7–44.7)	<0.0001
CPK, U/L	38–174	62.0 (47.0–90.0)	53.0 (36.5–79.0)	0.157	78.0 (55.0–128.5)	68.0 (48.0–105.5)	0.694	94.0 (60.0–148.0)	60.0 (44.0–89.0)	0.095
CK–MB, U/L	0–25	55.8 (34.9–77.1)	32.4 (27.9–54.8)	<0.0001	21.9 (13.1–45.7)	24.1 (11.7–52.7)	0.011	13.3 (10.6–15.7)	10.7 (8.4–13.4)	<0.0001
LDH, U/L	80–285	366.0 (225.5–530.0)	277.0 (174.5–357.0)	<0.0001	264.0 (182.0–361.5)	222.0 (169.0–294.5)	<0.0001	201.0 (177.0–247.0)	177.0 (159.0–211.0)	<0.0001
Potassium, mmol/L	3.5–5.3	4.2 (3.7–4.6)	4.1 (3.8–4.4)	0.394	3.8 (3.5–4.3)	4.1 (3.7–4.4)	0.001	3.7 (3.4–4.1)	4.1 (3.8–4.4)	<0.0001
Sodium, mmol/L	137–147	142.7 (137.1–147.1)	142.1 (138.2–145.3)	0.241	138.2 (134.7–141.4)	139.6 (137.2–143.1)	<0.0001	137.0 (135.0–139.0)	140.0 (139.0–142.0)	<0.0001
Total calcium, mmol/L	2.08–2.6	1.8 (1.5–2.0)	1.9 (1.6–2.0)	0.003	2.1 (2.0–2.3)	2.1 (2.0–2.2)	0.383	2.3 (2.2–2.4)	2.3 (2.2–2.3)	0.022

p-values denote the significance of differences in each parameter acquired upon hospital admission and upon hospital discharge in the same cohort of patients.

## Discussion

With 95% identity in its *S* gene to SARS-CoV, SARS-CoV-2 oriented its receptor-binding domain (RBD) and optimized its conformation to secure ACE2 in the host for cell entry, following the same manner as SARS-CoV but with higher affinity (18). ACE2 was known as a vasoconstrictive protein that regulated the renal and cardiovascular function, expressed in the pneumocytes of lung epithelia and enterocytes of the small intestine (19). This may explain why in addition to the predominant respiratory or pulmonary manifestation, the gastrointestinal symptoms occurred in a substantial portion of patients with mild COVID-19 in our study, including abdominal pain, diarrhea, or vomiting, consistent with other reports (20). In contrast, the delta variant of SARS-CoV-2 owns prominent mutations in its S protein, accounting for its increased infectivity and elevated capacity to escape immune recognition (21). To give a glimpse, the reproductive number (*R*_0_) for SARS-CoV-2 was 2.79, while the mean *R*_0_ of its delta variant reached a value of 5.08 (22). Differently, influenza viruses use hemagglutinins (HAs) and neuraminidases (NAs) on their surface to bind sialic acids (SAs) as receptors on the host cells for viral invasion, while SAs are ubiquitous in a broad spectrum of human cells (23). The median *R*_0_ value for seasonal influenza was estimated to be 1.28 (24). Altogether, a diversity of virological features, cellular tropism, and host specificity explains the variety in clinical profiles of different infections.

Previously, male patients and the elderly group were found susceptible to SARS-CoV-2 (25, 26). Our study here indicated a minimal difference between genders of patients with COVID-19, either infected with the wild-type or delta variant SARS-CoV-2, but a much higher male ratio in patients with seasonal influenza. The fact that men could become more prone to contract diverse viruses may be associated with non-gender factors, such as a smoking habit or occupational exposure to the pathogen (27). However, once infected, the male gender could be a risk factor for disease severity and mortality, where different degrees of inflammatory response could be induced by male or female patients to influence the disease course and outcome (27, 28). Furthermore, our study here confirmed that elderliness posed a high risk for SARS-CoV-2 infection. Many have concluded this to the changing ACE2 activity with aging, where the specific binding of ACE2 to virus outweighs the protective functions of ACE2 to major organs (29). However, whether and how ACE2 activity varies over age have yet been confirmed. This age predisposition became even more in patients with influenza. One explanation to answer why seniors have a greater susceptibility to infectious diseases than younger adults could be attributed to age-related immune dysfunction with concomitant chronic disorders (30).

Our study agreed with others in that hypertension, diabetes, bronchitis, and cardiovascular diseases made the leading comorbidities succumb to COVID-19 (25, 31). Hypertension, diabetes, and cardiovascular diseases ranked the three leading comorbidities in patients infected with the delta variant SARS-CoV-2, followed by bronchitis, which could be tied to the lesser portion of patients with smoking history in this cohort. Reportedly, people with chronic pulmonary diseases (e.g., bronchitis), cardiovascular diseases, and diabetes are known as high-risk groups for influenza illness, in line with our findings here (32, 33). Nevertheless, our finding that hypertension is the top comorbidity among patients with seasonal influenza may be ascribed to specific Chinese ethnicity studied where hypertension prevails (34).

Patients with COVID-19 were found to have a higher viral load in the nasal swabs or sputum samples than in the throat swabs (35). Moreover, SARS-CoV-2 was inclined to infect the lower airway, including the trachea, the bronchi, and the alveoli (26). This agreed with our findings where involuntary cough was the most common symptom in patients with COVID-19, followed by febrile illness as a sign of infection. Compared to the wild-type SARS-CoV-2 detected in the patients, the delta variant showed a much higher viral load and a longer period of viral shedding (36). Those results pointed out that in the quest to prevent COVID-19 contraction and stop SARS-CoV-2 spreading, oronasal covering and social distancing in addition to timely vaccination are still imperative measures to thwart the otherwise respiratory tract transmission inter-personally, or it would become harder to contain the virus for avoiding further deep lung infection.

Oronasal entry of respiratory virus led to its direct infection in the pulmonary system, as well as earning a chance to enter the bloodstream and then contract extrapulmonary organs through blood flow. Upon hospital admission, our clinical data from a substantial portion of patients with COVID-19 or influenza showed the abnormality in several blood parameters, including peripheral blood cells, hepatic enzymes, renal metabolites, and myocardial proteins, indicating acute assaults to immune systems together with damages to major organs including liver, kidney, and heart. Our results here were in concert with previous reports (1, 11, 23, 31).

Of note, electrolyte disorders have been found frequently in patients with COVID-19, including hypokalemia, hyponatremia, and hypocalcemia (37–41). ACE2, a key role in the renin-angiotensin system (RAS), converts angiotensin II into angiotensin-(1-7), a process that modulates the vasoconstriction and renal reabsorption (29). Therefore, ACE2 binding by SARS-CoV-2 might downregulate its expression, negatively affecting the electrolyte balance in the body fluid. Our study here reported many patients with mild COVID-19 experienced traces of hypokalemia and hyponatremia and most of them showed hypocalcemia. Hypokalemia was a result of continuous potassium loss in the urine of patients with COVID-19, following the degradation of ACE2 by SARS-CoV-2 and the disruption of the RAS system (40). Independently, hyponatremia was inversely correlated with the IL-6 increase in serum, indicating renal insufficiency and predicting the poor outcome in patients with COVID-19 (39, 41). In addition, hypocalcemia appeared frequently in patients with COVID-19, in correlation with increased inflammatory responses, elevated D-dimer levels, aggravated vitamin D deficiency, and worsened patient outcomes (37, 38). In parallel, electrolyte disorders were also commonly observed in patients with seasonal influenza. Although the low intake of electrolytes in viral infections could be multifactorial, it might be linked to some clinical characteristics of patients with COVID-19 or influenza, including myocardial injuries (40).

Regardless of previously underlying diseases, a considerable amount of cardiac injury in patients with COVID-19 has been noticed with a correlation to disease severity and mortality (42, 43). Myocardial biomarkers were considered prognostic factors for COVID-19 outcome through a systematic review (44). Upon hospital admission, COVID-19 patients with cardiovascular diseases but with normal troponin T levels were found a more favorable prognosis when compared to COVID-19 patients with elevated troponin T levels but without cardiovascular diseases (43). Therefore, myocardial injury developed along the course of COVID-19 and could worsen as the severity of COVID-19 aggravated, while inflammation was a potential trigger for myocardial impairment (43). For the same reason, acute myocardial infarction, fulminant myocarditis, and cardiac death could be associated with heightened risks of COVID-19 mortality (45, 46). Similarly, influenza viruses have also been reported to cause myocardial and cardiac injuries through direct infection of the heart and/or indirect induction of cytokines, associated with an increased risk of mortality (47). Seasonal influenza infection had been found consistently peaked along with wintertime cardiovascular mortality (32). Those explained the abnormal myocardial biomarkers observed in even patients with mild influenza. However, the myocardial injury and recovery in patients with COVID-19 and influenza differed due to the differing viremic effects on myocardial epithelium and the secondary effects on other cells/organs. For instance, coagulation dysfunction and vascular thrombosis induced by SARS-CoV-2 could be distinguished from those induced by the influenza virus (23, 48).

Our study has some limitations. First, a small pool of clinical data from 245 patients with COVID-19, 377 patients with seasonal influenza, and 115 patients with COVID-19 infected by the delta variant of SARS-CoV-2 were included here. To establish a relationship between clinical data and their primary outcome, a large dataset is required to minimize the influence of non-representative subjects and biased cases. For this reason, later analyses by adopting as many clinical features as possible from different hospitals and even different countries could cast a more comprehensive view on comparisons between two infectious diseases induced by respiratory viruses. Second, due to the emergency nature of COVID-19 as an emerging and devastating disease, only a limited set of laboratory tests was available for patients included in our study, and these blood parameters were not continuously monitored in the following course of disease development. A multi-angle and time-dynamic view on comparison between COVID-19 and seasonal influenza could have been otherwise obtained.

## Conclusion

With no specific treatment, early intervention using broad-spectrum antibiotics and antiviral drugs partially reversed the viral insults in patients with mild COVID-19 and influenza, although the treatment was far from satisfying. Therefore, when facing the flu season amid the COVID-19 pandemic, vaccinations of both flu and COVID-19 jabs should be reinforced, along with rapid identification of SARS-CoV-2 and its changing variants, close monitoring of COVID-19 positive population, and timely viral therapeutics with effectiveness.

## Data availability statement

The raw data supporting the conclusions of this article will be made available by the authors, without undue reservation.

## Ethics statement

The study was approved by Ethics Commission of The First People's Hospital of Jiangxia District (TFPHJD) in Wuhan, The Third People's Hospital of Yangzhou City (TTPHYC), and The Affiliated Hospital of Jiangsu University (TAHJU) in Zhenjiang, China, respectively. Written informed consent for participation was not required for this study in accordance with the national legislation and the institutional requirements.

## Author contributions

ZT conceived the idea and designed the study. JZ, XH, and ZT had approved access to data in the study and contributed to the writing of the manuscript. XH and ZT contributed to the statistical analysis. All authors reviewed and approved the manuscript submission.

## Conflict of interest

The authors declare that the research was conducted in the absence of any commercial or financial relationships that could be construed as a potential conflict of interest.

## Publisher's note

All claims expressed in this article are solely those of the authors and do not necessarily represent those of their affiliated organizations, or those of the publisher, the editors and the reviewers. Any product that may be evaluated in this article, or claim that may be made by its manufacturer, is not guaranteed or endorsed by the publisher.

## References

[1] Huang C Wang Y Li X Ren L Zhao J Hu Y. Clinical features of patients infected with 2019 novel coronavirus in Wuhan, China. Lancet. (2020) 395:497–506. 10.1016/S0140-6736(20)30183-5 31986264 PMC7159299

[2] Zhu N Zhang D Wang W Li X Yang B Song J. A novel coronavirus from patients with pneumonia in China, 2019. N Engl J Med. (2020) 382:727–33. 10.1056/NEJMoa2001017 31978945 PMC7092803

[3] Jiang S Shi Z Shu Y Song J Gao GF Tan W. A distinct name is needed for the new coronavirus. Lancet. (2020) 395:949. 10.1016/S0140-6736(20)30419-0 32087125 PMC7124603

[4] Wu Y Ho W Huang Y Jin DY Li S Liu SL. SARS-CoV-2 is an appropriate name for the new coronavirus. Lancet. (2020) 395:949–50. 10.1016/S0140-6736(20)30557-2 32151324 PMC7133598

[5] Organization WH. COVID-19 Weekly Epidemiological Update. 97 ed. Geneva: WHO (2022).

[6] Zhou P Yang XL Wang XG Hu B Zhang L Zhang W. A pneumonia outbreak associated with a new coronavirus of probable bat origin. Nature. (2020) 579:270–3. 10.1038/s41586-020-2012-7 32015507 PMC7095418

[7] Ren LL Wang YM Wu ZQ Xiang ZC Guo L Xu T. Identification of a novel coronavirus causing severe pneumonia in human: a descriptive study. Chin Med J. (2020) 133:1015–24. 10.1097/CM9.0000000000000722 32004165 PMC7147275

[8] Hosseiny M Kooraki S Gholamrezanezhad A Reddy S Myers L. Radiology perspective of coronavirus disease 2019 (COVID-19): lessons from severe acute respiratory syndrome and middle east respiratory syndrome. Am J Roentgenol. (2020) 2020:1–5. 10.2214/AJR.20.22969 32108495

[9] Karim SSA Karim QA. Omicron SARS-CoV-2 variant: a new chapter in the COVID-19 pandemic. Lancet. (2021) 398:2126–8. 10.1016/S0140-6736(21)02758-6 34871545 PMC8640673

[10] Hoffmann M Kleine-Weber H Schroeder S Kruger N Herrler T Erichsen S. SARS-CoV-2 cell entry depends on ACE2 and TMPRSS2 and is blocked by a clinically proven protease inhibitor. Cell. (2020) 181:271–80 e8. 10.1016/j.cell.2020.02.052 32142651 PMC7102627

[11] Zhang J Ding D Huang X Zhang J Chen D Fu P. Differentiation of COVID-19 from seasonal influenza: a multicenter comparative study. J Med Virol. (2021) 93:1512–9. 10.1002/jmv.26469 32856744 PMC7461066

[12] Groves HE Papenburg J Mehta K Bettinger JA Sadarangani M Halperin SA. The effect of the COVID-19 pandemic on influenza-related hospitalization, intensive care admission and mortality in children in Canada: a population-based study. Lancet Region Health Americas. (2022) 7:100132. 10.1016/j.lana.2021.100132 35291567 PMC8913102

[13] McCauley J Barr IG Nolan T Tsai T Rockman S Taylor B. The importance of influenza vaccination during the COVID-19 pandemic. Influenza Other Respir Viruses. (2022) 16:3–6. 10.1111/irv.12917 34605171 PMC8652850

[14] Rubin R. Influenza's unprecedented low profile during COVID-19 pandemic leaves experts wondering what this flu season has in store. J Am Med Assoc. (2021) 326:899–900. 10.1001/jama.2021.14131 34431979

[15] Zhang J Huang X Ding D Zhang J Xu L Hu Z. Comparative study of acute lung injury in COVID-19 and non-COVID-19 patients. Front Med. (2021) 8:666629. 10.3389/fmed.2021.666629 34485324 PMC8415545

[16] China NHCo. Guidelines for the Diagnosis and Treatment of Novel Coronavirus (2019-nCoV) Infection by the National Health Commission (Trial Version 7). Beijing: National Health Commission of China (2020).

[17] Xu Y Chen Y Tang X. Guidelines for the diagnosis and treatment of coronavirus disease 2019 (COVID-19) in China. Glob Health Med. (2020) 2:66–72. 10.35772/ghm.2020.01015 33330780 PMC7731342

[18] Wrapp D Wang N Corbett KS Goldsmith JA Hsieh CL Abiona O. Cryo-EM structure of the 2019-nCoV spike in the prefusion conformation. Science. (2020) 367:1260–3. 10.1126/science.abb2507 32075877 PMC7164637

[19] Hamming I Timens W Bulthuis ML Lely AT Navis G van Goor H. Tissue distribution of ACE2 protein, the functional receptor for SARS coronavirus: a first step in understanding SARS pathogenesis. J Pathol. (2004) 203:631–7. 10.1002/path.1570 15141377 PMC7167720

[20] Jin X Lian JS Hu JH Gao J Zheng L Zhang YM. Epidemiological, clinical and virological characteristics of 74 cases of coronavirus-infected disease 2019 (COVID-19) with gastrointestinal symptoms. Gut. (2020) 69:1002–9. 10.1136/gutjnl-2020-320926 32213556 PMC7133387

[21] Khateeb J Li Y Zhang H. Emerging SARS-CoV-2 variants of concern and potential intervention approaches. Crit Care. (2021) 25:244. 10.1186/s13054-021-03662-x 34253247 PMC8274962

[22] Liu Y Rocklöv J. The reproductive number of the Delta variant of SARS-CoV-2 is far higher compared to the ancestral SARS-CoV-2 virus. J Travel Med. (2021) 2021:taab124. 10.1093/jtm/taab124 34369565 PMC8436367

[23] Zhang J Huang X Ding D Tao Z. Platelet-driven coagulopathy in COVID-19 patients: in comparison to seasonal influenza cases. Exp Hematol Oncol. (2021) 10:34. 10.1186/s40164-021-00228-z 34059141 PMC8165133

[24] Biggerstaff M Cauchemez S Reed C Gambhir M Finelli L. Estimates of the reproduction number for seasonal, pandemic, and zoonotic influenza: a systematic review of the literature. BMC Infect Dis. (2014) 14:480. 10.1186/1471-2334-14-480 25186370 PMC4169819

[25] Zhou F Yu T Du R Fan G Liu Y Liu Z. Clinical course and risk factors for mortality of adult inpatients with COVID-19 in Wuhan, China: a retrospective cohort study. Lancet. (2020) 395:1054–62. 10.1016/S0140-6736(20)30566-3 32171076 PMC7270627

[26] Guan WJ Ni ZY Hu Y Liang WH Ou CQ He JX. Clinical characteristics of coronavirus disease 2019 in China. N Engl J Med. (2020) 382:1708–20. 10.1056/NEJMoa2002032 32109013 PMC7092819

[27] Morgan R Klein SL. The intersection of sex and gender in the treatment of influenza. Curr Opin Virol. (2019) 35:35–41. 10.1016/j.coviro.2019.02.009 30901632 PMC6556398

[28] Peckham H de Gruijter NM Raine C Radziszewska A Ciurtin C Wedderburn LR. Male sex identified by global COVID-19 meta-analysis as a risk factor for death and ITU admission. Nat Commun. (2020) 11:6317. 10.1038/s41467-020-19741-6 33298944 PMC7726563

[29] Hamming I Cooper ME Haagmans BL Hooper NM Korstanje R Osterhaus AD. The emerging role of ACE2 in physiology and disease. J Pathol. (2007) 212:1–11. 10.1002/path.2162 17464936 PMC7167724

[30] Yoshikawa TT. Epidemiology and unique aspects of aging and infectious diseases. Clin Infect Dis. (2000) 30:931–3. 10.1086/313792 10880303

[31] Wang D Hu B Hu C Zhu F Liu X Zhang J. Clinical characteristics of 138 hospitalized patients with 2019 novel coronavirus-infected pneumonia in Wuhan, China. J Am Med Assoc. (2020) 323:1061–9. 10.1001/jama.2020.1585 32031570 PMC7042881

[32] Nguyen JL Yang W Ito K Matte TD Shaman J Kinney PL. Seasonal influenza infections and cardiovascular disease mortality. J Am Med Assoc Cardiol. (2016) 1:274–81. 10.1001/jamacardio.2016.0433 27438105 PMC5158013

[33] Uyeki TM. High-risk groups for influenza complications. J Am Med Assoc. (2020) 324:2334. 10.1001/jama.2020.21869 33136143

[34] Wang Z Chen Z Zhang L Wang X Hao G Zhang Z. Status of hypertension in China: results from the China Hypertension Survey, 2012-2015. Circulation. (2018) 137:2344–56. 10.1161/CIRCULATIONAHA.117.032380 29449338

[35] Pan Y Zhang D Yang P Poon LLM Wang Q. Viral load of SARS-CoV-2 in clinical samples. Lancet Infect Dis. (2020) 20:411–2. 10.1016/S1473-3099(20)30113-4 32105638 PMC7128099

[36] Wang Y Chen R Hu F Lan Y Yang Z Zhan C. Transmission, viral kinetics and clinical characteristics of the emergent SARS-CoV-2 Delta VOC in Guangzhou, China. EClinicalMedicine. (2021) 40:101129. 10.1016/j.eclinm.2021.101129 34541481 PMC8435265

[37] Di Filippo L Formenti AM Rovere-Querini P Carlucci M Conte C Ciceri F. Hypocalcemia is highly prevalent and predicts hospitalization in patients with COVID-19. Endocrine. (2020) 68:475–8. 10.1007/s12020-020-02383-5 32533508 PMC7292572

[38] Di Filippo L Doga M Frara S Giustina A. Hypocalcemia in COVID-19: prevalence, clinical significance and therapeutic implications. Rev Endocr Metab Disord. (2021) 23:299–308. 10.1007/s11154-021-09655-z 33846867 PMC8041474

[39] Akbar MR Pranata R Wibowo A Irvan A Sihite TA Martha JW. The prognostic value of hyponatremia for predicting poor outcome in patients with COVID-19: a systematic review and meta-analysis. Front Med. (2021) 8:666949. 10.3389/fmed.2021.666949 34195209 PMC8236602

[40] Chen D Li X Song Q Hu C Su F Dai J. Assessment of hypokalemia and clinical characteristics in patients with coronavirus disease 2019 in Wenzhou, China. J Am Med Assoc Netw Open. (2020) 3:e2011122. 10.1001/jamanetworkopen.2020.11122 32525548 PMC7290402

[41] Hu W Lv X Li C Xu Y Qi Y Zhang Z. Disorders of sodium balance and its clinical implications in COVID-19 patients: a multicenter retrospective study. Intern Emerg Med. (2021) 16:853–62. 10.1007/s11739-020-02515-9 33064253 PMC7563904

[42] Shi S Qin M Shen B Cai Y Liu T Yang F. Association of cardiac injury with mortality in hospitalized patients with COVID-19 in Wuhan, China. J Am Med Assoc Cardiol. (2020) 5:802–10. 10.1001/jamacardio.2020.0950 32211816 PMC7097841

[43] Guo T Fan Y Chen M Wu X Zhang L He T. Cardiovascular implications of fatal outcomes of patients with coronavirus disease 2019 (COVID-19). J Am Med Assoc Cardiol. (2020) 5:811–8. 10.1001/jamacardio.2020.1017 32219356 PMC7101506

[44] Izcovich A Ragusa MA Tortosa F Lavena Marzio MA Agnoletti C Bengolea A. Prognostic factors for severity and mortality in patients infected with COVID-19: a systematic review. PLoS ONE. (2020) 15:e0241955. 10.1371/journal.pone.0241955 33201896 PMC7671522

[45] Solomon MD McNulty EJ Rana JS Leong TK Lee C Sung SH. The covid-19 pandemic and the incidence of acute myocardial infarction. N Engl J Med. (2020) 383:691–3. 10.1056/NEJMc2015630 32427432

[46] Ni W Yang X Liu J Bao J Li R Xu Y. Acute myocardial injury at hospital admission is associated with all-cause mortality in COVID-19. J Am Coll Cardiol. (2020) 76:124–5. 10.1016/j.jacc.2020.05.007 32407771 PMC7213968

[47] Gao C Wang Y Gu X Shen X Zhou D Zhou S. Association between cardiac injury and mortality in hospitalized patients infected with avian influenza A (H7N9) virus. Crit Care Med. (2020) 48:451. 10.1097/CCM.0000000000004207 32205590 PMC7098447

[48] Khan MS Shahid I Anker SD Solomon SD Vardeny O Michos ED. Cardiovascular implications of COVID-19 versus influenza infection: a review. BMC Med. (2020) 18:403. 10.1186/s12916-020-01816-2 33334360 PMC7746485

